# IK‐5f targets amyloid aggregates and alleviates cognitive deficits in the 5XFAD Alzheimer mouse model

**DOI:** 10.1002/alz70859_101506

**Published:** 2025-12-25

**Authors:** Heewon Shin, Sunhee Lee, Seok Hyun Yoon, Illhwan Cho, InWook Park, Soljee Yoon, Suhyun Ye, Songmin Lee, Ikyon Kim, YoungSoo Kim

**Affiliations:** ^1^ Yonsei University, Incheon, Incheon Korea, Republic of (South); ^2^ Yonsei Institute of Pharmaceutical Sciences, Incheon, Incheon Korea, Republic of (South); ^3^ Yonsei University, Yeonsu‐gu, Incheon Korea, Republic of (South); ^4^ Yonsei Institute of Pharmaceutical Sciences, Yeonsu‐gu, Incheon Korea, Republic of (South)

## Abstract

**Background:**

Alzheimer's disease (AD) involves progressive synaptic dysfunction and extracellular deposition of amyloid‐β (Aβ) plaques. Current immunotherapies targeting Aβ are effective but associated with amyloid‐related imaging abnormalities. Small‐molecule drugs that disaggregate Aβ aggregates may provide a safer, cost‐effective alternative.

**Method:**

Six novel chemical compounds were synthesized and screened for anti‐Aβ activity using Thioflavin T fluorescence assays. IK‐5f, the most effective compound, was evaluated in 5XFAD mice via a four‐week intraperitoneal injection regimen. Cognitive performance was assessed using the Y‐maze test, and Aβ plaques and neuroinflammation were analyzed through immunohistochemical staining and dot blot assays.

**Result:**

IK‐5f significantly inhibited Aβ aggregation and promoted fibril dissociation in vitro. In 5XFAD mice, IK‐5f improved cognitive performance in the Y‐maze test, reduced hippocampal Aβ plaque burden, and decreased neuroinflammation, as evidenced by reduced GFAP staining and 6E10‐immunoreactive aggregates.

**Conclusion:**

IK‐5f exhibits strong potential as a small‐molecule drug candidate for AD by effectively reducing Aβ pathology and neuroinflammation while enhancing cognitive function.

